# Credit to Whom Credit Is Due

**Published:** 1871-06

**Authors:** 


*Credit to whom Credit is due.*

In the April number of this Journal, we unintentionally omitted to credit the Baltimore (Maryland) Journal with an article copied from it, headed "Extraordinary Case of Enlarged Spleen." Such oversights will sometimes occur, and we hope our Baltimore friend will excuse our negligence, on that occasion.

